# Large-scale identification of odorant-binding proteins and chemosensory proteins from expressed sequence tags in insects

**DOI:** 10.1186/1471-2164-10-632

**Published:** 2009-12-25

**Authors:** Ya-Long Xu, Peng He, Lan Zhang, Shao-Qing Fang, Shuang-Lin Dong, Yong-Jun Zhang, Fei Li

**Affiliations:** 1 Department of Entomology, Nanjing Agricultural University/Key Laboratory of Monitoring and Management of Crop Diseases and Pest Insects, Ministry of Agriculture, Nanjing 210095, PR China; 2 Yantai Entry-exit Inspection and Quarantine Bureau, Yantai 264000, Shandong, PR China; 3 State Key Laboratory for Biology of Plant Disease and Insect Pests, Institute of Plant Protection, Chinese Academy of Agricultural Sciences, Beijing 100094, PR China

## Abstract

**Background:**

Insect odorant binding proteins (OBPs) and chemosensory proteins (CSPs) play an important role in chemical communication of insects. Gene discovery of these proteins is a time-consuming task. In recent years, expressed sequence tags (ESTs) of many insect species have accumulated, thus providing a useful resource for gene discovery.

**Results:**

We have developed a computational pipeline to identify OBP and CSP genes from insect ESTs. In total, 752,841 insect ESTs were examined from 54 species covering eight Orders of Insecta. From these ESTs, 142 OBPs and 177 CSPs were identified, of which 117 OBPs and 129 CSPs are new. The complete open reading frames (ORFs) of 88 OBPs and 123 CSPs were obtained by electronic elongation. We randomly chose 26 OBPs from eight species of insects, and 21 CSPs from four species for RT-PCR validation. Twenty two OBPs and 16 CSPs were confirmed by RT-PCR, proving the efficiency and reliability of the algorithm. Together with all family members obtained from the NCBI (OBPs) or the UniProtKB (CSPs), 850 OBPs and 237 CSPs were analyzed for their structural characteristics and evolutionary relationship.

**Conclusions:**

A large number of new OBPs and CSPs were found, providing the basis for deeper understanding of these proteins. In addition, the conserved motif and evolutionary analysis provide some new insights into the evolution of insect OBPs and CSPs. Motif pattern fine-tune the functions of OBPs and CSPs, leading to the minor difference in binding sex pheromone or plant volatiles in different insect Orders.

## Background

Insects are highly successful terrestrial animals that have complicated communication systems. Insect odorant binding proteins (OBPs) play an important role in insect chemical communication. Until recently, it was believed that pheromones and other odors entering the aqueous lumen of chemosensilla, were transported by OBPs to transmembrane odorant receptors (ORs) [1,2] and finally degraded by odorant degradation enzymes (ODEs) [3-7]. Recently, however, an active role of OBPs has been reported, where a conformational change of the OBP triggered by the presence of the ligand in its binding pocket that activated the membrane-bound receptor [8]. Insect OBPs, particularly in Lepidoptera, can be classified into two subfamilies, pheromone-binding proteins (PBPs) and general odorant binding proteins (GOBPs)[9]. OBPs are small and water soluble proteins 120 to 150 amino acids long. A typical feature of OBPs is the presence of six positional conserved cysteines. These six cysteines form three disulfide bridges, which play important roles in maintaining the protein tertiary structure. Another essential criterion is an acceptable similarity in protein sequence (e-value of BLAST analysis) with other family members. Insect chemosensory proteins (CSPs) represent another gene family suggested to have similar properties in binding and transporting pheromones and other ligands. Insect CSPs are smaller than OBPs with about 100-120 amino acids, and bear no sequence similarity with OBPs. CSPs have only four conserved cysteines linked by disulfide bridges between neighboring residues [10] and are better conserved than OBPs across species [11].

Numerous efforts have been made to obtain the sequences of insect OBPs [9,12-20] and CSPs [14,21-28] by direct cloning, which normally involves designing degenerate primers based on conserved protein sequences, amplifying the fragment and obtaining the full length sequences by Rapid Amplification of cDNA Ends (RACE). Thanks to the accomplishments of genome sequencing projects of several insect species, large scale new gene discovery is possible using bioinformatics. By searching available genome sequences, Hekmat-Scafe et al. found 51 OBP genes in *Drosophila melanogaster *and a new subfamily of OBPs [29]; Maleszka et al. showed that *Apis mellifera *has only 21 OBP genes[14]; Zhou et al. identified 66 putative OBPs in *Aedes aegypti *and 11 additional sequences in *Anopheles gambiae *by developing a specific algorithm [30]. By comparative genomic analysis of the OBP families in 12 *Drosophila *genomes, Vieira et al. identified 595 OBP genes and found that purifying selection governs the evolution of the OBP family [31]. In 2006, Zhou et al. did a comprehensive searching for CSP genes from insect genomes and ESTs and identified 74 putative CSP genes from 22 insect species[32]. Gong et al. performed a genome-wide analysis based on the conserved cysteine residues and similarity to CSPs in other insects, finding 20 candidate CSPs in the silkworm [33]. However, genome searching for new genes is limited to a few insect species, as genome sequences are not available for most insects. Fortunately, an increasing number of insect expressed sequence tags (ESTs) are deposited in the dbEST database of the National Center for Biotechnology Information (NCBI). Insect ESTs are a valuable resource that has not been fully exploited for mining new OBP or CSP genes. Pugalenthi et al. developed a new algorithm using Regularized Least Squares Classifier (RLSC) to predict OBPs with a high accuracy of 97.7%. This approach could be used to identify novel OBPs that have low similarities with known ones [34]. Recently, Zhou et al. used MotifSearch algorithm to screen putative OBPs in the silkworm and found 13 OBP-like genes, which is much fewer than that in fruit flies and mosquitoes[35].

Here, we develop a computational pipeline to identify OBP and CSP genes from insect ESTs of 54 species across eight Orders including Blattaria, Coleoptera, Diptera, Hemiptera, Hymenoptera, Lepidoptera, Orthoptera and Phthiraptera. In total, 117 new OBPs and 129 new CSPs were found, of which 38 genes from eight species were experimentally validated by RT-PCR. In addition, the conserved cysteines patterns, motif patterns and phylogenetic relationship of known OBPs and CSPs were analyzed.

## Results

### Identification of new OBPs and CSPs genes from insect ESTs

**Figure 1:**
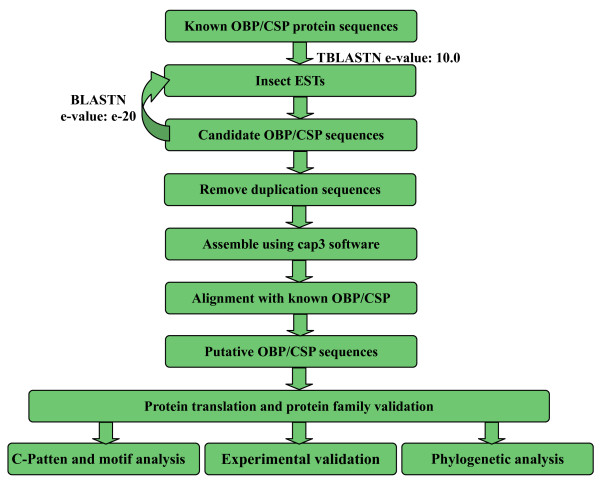
**The computational pipeline used to identify insect OBPs and CSPs from expressed sequence tags**. The accession numbers of OBP and CSPs used in this analysis were listed in Additional File 6.

We collected 752,841 insect ESTs from the dbEST [36] and constructed a local database for further analysis. The ESTs are from 54 insect species that cover eight Orders of Insecta. We searched for OBPs and CSPs with a computational pipeline as detailed in Figure 1. In total, 2,380 ESTs were found to satisfy the strict criteria, and produce 142 OBPs from 38 species and 177 CSPs from 37 species. Of these genes, more than 80% OBPs (117) and 70% CSPs (129) have not been reported before (Table 1, Additional File 1). We performed electronic elongation to get the sequences as long as possible and obtained the intact ORFs of 88 OBPs and 123 CSPs. The nucleotide and protein sequences of predicted OBPs are listed in Additional File 2 and 3, and those of predicted CSPs in Additional File 4 and 5.

**Table 1:** The OBPs and CSPs predicted from insect ESTs

Orders	Species	OBP_number	CSP_number	Total EST number
**Blattaria**	*Periplaneta americana*	3(3)	4(3)	2550
**Coleoptera**	*Diabrotica virgifera virgifera*	29(29)	8(8)	17782
	*Diaprepes abbreviatus*	3(3)	1(1)	5219
	*Hypothenemus hampei*	2(2)	0	2032
	*Leptinotarsa decemlineata*	4(4)	2(2)	8410
**Diptera**	*Anopheles funestus*	0	3(3)	2846
	*Chironomus tentans*	0	1(1)	8584
	*Culicoides sonorensis*	3(0)	0	2977
	*Glossina morsitans*	1(1)	0	41799
	*Haematobia irritans irritans*	6(6)	0	16916
	*Lutzomyia longipalpis*	1(1)	1(1)	21069
	*Mayetiola destructor*	1(1)	0	1803
	*Oncometopia nigricans*	3(3)	3(3)	9065
	*Phlebotomus papatasi*	2(2)	1(1)	36583
	*Rhynchosciara americana*	1(1)	0	8581
	*Orseolia oryzae*	0	0	1259
	*Sitodiplosis mosellana*	1(1)	0	1217
**Hemiptera**	*Acyrthosiphon pisum*	4(4)	10(4)	167706
	*Aphis gossypii*	1	4(4)	8344
	*Bemisia tabaci*	0	1(1)	9110
	*Diaphorina citri*	1	2(2)	5906
	*Graphocephala atropunctata*	2(2)	4(4)	6481
	*Homalodisca coagulata*	4(4)	5(5)	20030
	*Myzus persicae*	4(4)	5(3)	27687
	*Nilaparvata lugens*	3(3)	9(9)	37311
	*Rhodnius prolixus*	4(4)	2(2)	10196
	*Oncopeltus fasciatus*	0	0	1115
	*Toxoptera citricida*	2(2)	0	4304
**Hymenoptera**	*Lysiphlebus testaceipes*	7(7)	4(4)	7840
	*Nasonia giraulti*	10(10)	8(8)	30060
	*Nasonia vitripennis*	3	2	12354
	*Solenopsis invicta*	8(7)	15(14)	22850
	*Microctonus hyperodae*	0	0	1104
	*Vespula squamosa*	3(3)	4(4)	2144
**Lepidoptera**	*Agrotis segetum*	2(1)	7(7)	2286
	*Bicyclus anynana*	0	5(5)	10159
	*Danaus plexippus*	1(1)	2(2)	19577
	*Heliconius melpomene*	0	6(3)	4971
	*Lonomia obliqua*	1(1)	5(3)	1503
	*Manduca sexta*	14	12	3317
	*Heliconius erato/himera mixed*	1	0	9394
	*Plutella xylostella*	0	1	1134
	*Plodia interpunctella*	1(1)	2(2)	6234
	*Spodoptera frugiperda*	0	2(1)	32330
	*Antheraea mylitta*	0	3(3)	3888
	*Helicoverpa armigera*	0	0	1055
	*Heliothis virescens*	0	0	5340
	*Ostrinia nubilalis*	0	0	1742
	*Trichoplusia ni cabbage*	2(2)	1	12189
**Orthoptera**	*Gryllus bimaculatus*	3(3)	10(10)	11268
	*Laupala kohalensis*	0	4(4)	14377
	*Locusta migratoria*	1(1)	18(2)	45462
**Phthiraptera**	*Pediculus humanus capitis*	0	0	4508

**Total**	54	142(117)	177(129)	752841

The numbers in parenthesis are new OBPs or CSPs found in this paper. Nucleotide and protein sequences of these OBPs or CSPs are given in Additional File 2, 3, 4 &5.

In some insects, more than 10 OBPs or CSPs were identified. For example, 29 new OBPs were predicted in *Diabrotica virgifera *and 10 in *Nasonia giraulti*. Fifteen CSPs were predicted in *Solenopsis invicta*, of which 14 are not reported before, and 10 new CSPs genes were found in *Gryllus bimaculatus*. However, fewer than five OBPs or CSPs were identified in most species. We plotted the number of identified OBPs or CSPs against the total number of ESTs in each species and could not find any clear relationship (data not shown).

### Conserved cysteines pattern

The presence of conserved cysteines is a typical feature of OBPs and CSPs. We therefore analyzed the cysteines patterns (C-patterns) of OBPs and CSPs in different Orders (Table 2). Generally, there were no major differences between different Orders, except for the presence of a sub-class of OBPs, C-plus OBPs, in Diptera containing eight conserved cysteines. In the typical C-pattern, there were three amino acids between the second and third cysteines in all OBPs, while eight residues were present between the fifth and sixth cysteines in most insect OBPs. The numbers of amino acids between the other three neighboring cysteines were rather variable. In order to evaluate the variability in the distances between each pair of neighboring cysteines, we calculated the coefficients of variation (Table 3). In most insects, the distance between the fourth and the fifth cysteines was the most variable. However, in Hymenoptera, the distance between the first and the second cysteines was the most variable with a coefficient of variation of 11.66. The highest variations were found in the OBPs of Diptera. By contrast, C-patterns of CSPs were much more conserved.

**Table 2:** Conserved C-Pattern in OBPs and CSP

Protein	Order	Sequence number	C-Patten
**OBP**	Lepidoptera	68	C1-X_25-30_-C2-X_3_-C3-X_36-42_-C4-X_8-14_-C5-X_8_-C6
	Coleoptera	34	C1-X_23-44_-C2-X_3_-C3-X_36-43_-C4-X_8-12_-C5-X_8_-C6
		135	C1-X_21-68_-C2-X_3_-C3-X_21-46_-C4-X_8-28_-C5-X_8-9_-C6
	Diptera	41	C1-X_20-58_-C2-X_3_-C3-X_55-76_-C4-X_9_-C5-X_8_-C6-X_10-11_-C7-X_7-11_-C8
	Hemiptera	34	C1-X_22-32_-C2-X_3_-C3-X_36-46_-C4-X_8-14_-C5-X_8_-C6
	Hymenoptera	254	C1-X_23-35_-C2-X_3_-C3-X_27-45_-C4-X_7-14_-C5-X_8_-C6
	Orthoptera	4	C1-X_26-27_-C2-X_3_-C3-X_35-41_-C4-X_8-12_-C5-X_8_-C6
	
**CSP**	Coleoptera	26	C1-X_6-8_-C2-X_18_-C3-X_2_-C4
	Diptera	23	C1-X_6-8_-C2-X_18_-C3-X_2_-C4
	Hemiptera	34	C1-X_5-6_-C2-X_18-19_-C3-X_2_-C4
	Hymenoptera	31	C1-X_6-8_-C2-X_18-19_-C3-X_2_-C4
	Lepidoptera	87	C1-X_6_-C2-X_18_-C3-X_2_-C4
	Orthoptera	29	C1-X_6-8_-C2-X_18-19_-C3-X_2_-C4

**X: **Any amino acid. The C-pattern in Diptera includes two types (typical 6-C and atypical 8-C patterns), which are listed separately. The accession numbers of OBPs and CSPs used in this analysis were listed in Additional File 6.

**Table 3:** Coefficients of variation of the C-pattern in OBP and CSP genes

Protein	Order	Sequence number	Coefficient of variation				
			
			C1-C2	C2-C3	C3-C4	C4-C5	C5-C6
**OBP**	*Lepidoptera*	86	6.22	0	3.66	14.08	0
	*Coleoptera*	34	12.50	0	6.52	12.43	0
	*Diptera*	135	14.65	0	6.02	17.17	0.09
	*Hemiptera*	34	7.16	0	4.54	16.50	0
	*Hymenoptera*	254	11.66	0	3.75	8.10	0
	*Orthoptera*	4	1.90	0	6.71	20.16	0
	
**CSP**	*Coleoptera*	26	6.45	0	0	-	-
	*Diptera*	23	6.85	1.16	0	-	-
	*Hemiptera*	34	4.02	1.32	0	-	-
	*Hymenoptera*	31	9.70	2.69	0	-	-
	*Lepidoptera*	87	3.08	1.02	0	-	-
	*Orthoptera*	29	10.87	1.03	0	-	-

The coefficient of variation (CV) was calculated as Standard deviation divided by Means. In Diptera, the data for OBPs were calculated from OBPs of only 6-C pattern. The accession numbers of OBPs and CSPs used in this analysis were listed in Additional File 6.

### Motif-pattern analysis

**Figure 2:**
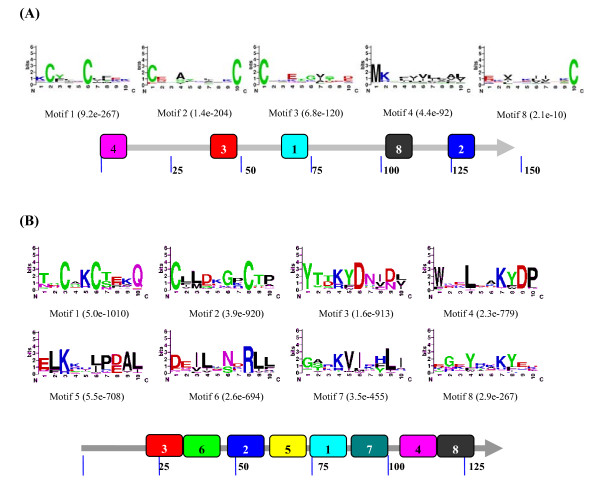
**Motif analysis of OBP (A) and CSP (B) genes**. Parameters used for motif discovery were: minimum width = 6, maximum = 10, maximum number of motif to find = 8. In each (A) and (B) panel, the upper parts are the motifs that existed in more than 50% investigated proteins. The lower parts indicate the approximate location of each motif on the protein sequence. The numbers in the boxes correspond to the numbered motifs in the upper part of the figure. The small number means high conservation. The locations of the motifs on the protein sequence are indicated with the amino acids numbers, starting from the N terminal. The accession numbers of OBP and CSPs used in this analysis were listed in Additional File 6.

The conserved motifs are important elements of functional domains. We used the MEME server to discover conserved motifs in OBPs and CSPs[37]. The full-length sequences of OBP and CSPs either collected from the database or newly predicted in this work were used for motif analysis. Parameters used in this and all other motif predictions of this study were: minimum width = 6, maximum = 10, maximum number of motif to find = 8. As a result, eight motifs were found for both CSPs and OBPs. Only five motifs were present in more than 50% of OBPs, while all eight motifs were present in more than 50% of CSPs (Figure 2).

**Figure 3:**
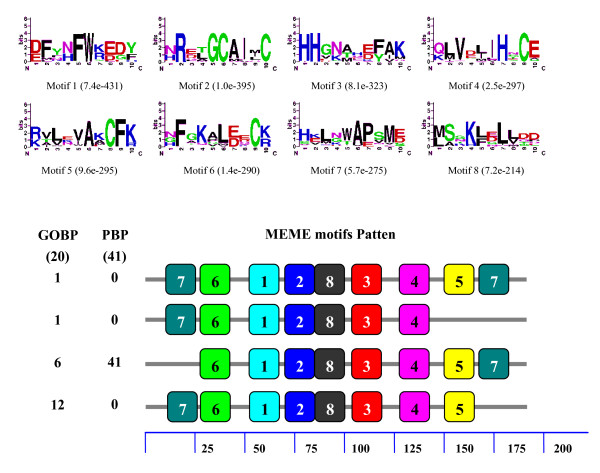
**Motif analysis of Lepidoptera PBPs and GOBPs**. The GOBPs and PBPs were combined into one set of sequences and then submitted to MEME server. Parameters used for motif discovery were: minimum width = 6, maximum = 10, maximum number of motif to find = 8. In the lower part of the figure, the numbers in the two columns on the left of the figure are the numbers of PBPs corresponding to the MEME motif patterns on the right. The numbers in the boxes correspond to the numbered motifs in the upper part of the figure. The small number means high conservation. The numbers on the bottom indicate approximate locations of each motif on the protein sequence, starting from the N terminal. The accession numbers of PBPs and GOBPs used in this analysis were listed in Additional File 6.

Since a high number of OBP genes have been reported in species of Lepidoptera, we carried out a motif-pattern analysis of GOBPs and PBPs to compare the differences between these two subfamilies. The GOBPs and PBPs were combined into one set of sequences and then submitted to MEME server. Although both GOBPs and PBPs have the same eight motifs, the motif-patterns were quite different (Figure 3). The seventh motif was located at the C-terminus of all 41 tested PBPs, but appeared at the N-terminus of 12 out of 20 GOBPs. Only six GOBPs shared the same motif-pattern with PBPs. Interestingly, one GOBP lacked the fifth motif and one had two copies of the seventh motif.

**Figure 4:**
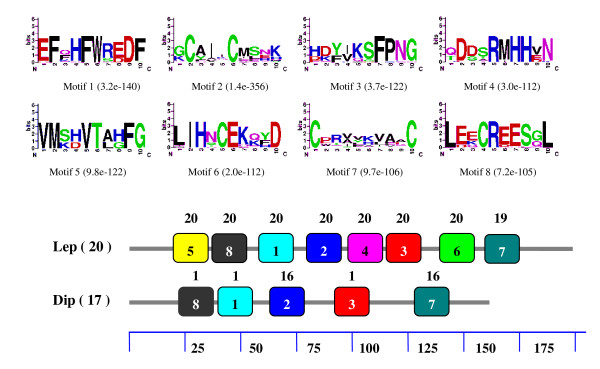
**Motifs analysis of Lepidoptera and Diptera GOBPs**. The GOBP sequences of both lepidopteran and dipteran were combined into a set of sequences for motif analysis. Parameters used for motif discovery were: minimum width = 6, maximum = 10, maximum number of motif to find = 8. In the lower part of the figure, the numbers in parenthesis indicate the total number of GOBPs investigated, of which the number of proteins possessing the motif is listed on the top of each motif with boxed number. The numbers in the boxes correspond to the numbered motifs in the upper part of the figure, where the smallest numbers indicate the highest conservation. The numbers on the bottom indicate approximate locations of each motif on the protein sequence, starting from the N terminal. The accession numbers of GOBPs used in this analysis were listed in Additional File 6.

**Figure 5:**
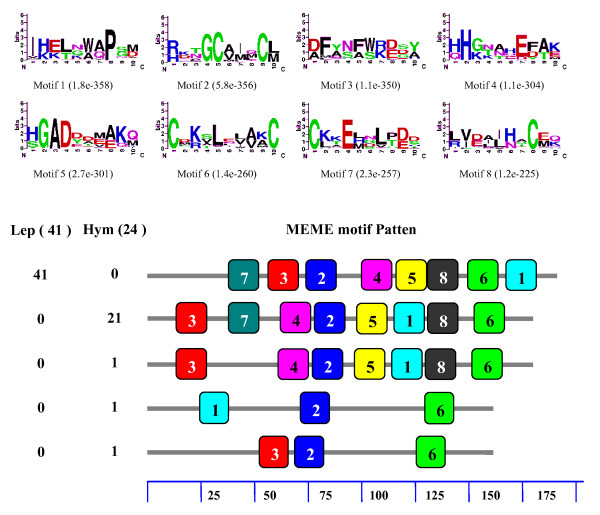
**Motifs analysis of Lepidoptera and Hymenoptera PBP**. The PBP sequences of both lepidopteran and dipteran were combined into a set of sequences for motif analysis. Parameters used for motif discovery were: minimum width = 6, maximum = 10, maximum number of motif to find = 8. In the lower part of the figure, the numbers in the two columns on the left are the numbers of PBPs corresponding to the MEME motif patterns on the right of the figure. The numbers in the boxes correspond to the numbered motifs in the upper part of the figure. The smallest number indicates the highest conservation. The numbers on the bottom indicate approximate locations of each motif on the protein sequence, starting from the N terminal. The accession numbers of PBPs used in this analysis were listed in Additional File 6.

When the GOBP sequences of both lepidopteran and dipteran were combined into a set of sequences for motif analysis, we also found that the motif patterns were different between lepidopteran and dipteran GOBPs (Figure 4). Of the eight motifs in the Lepidoptera, only the second and seventh were found in most dipteran GOBPs. The first, third and eighth motifs appeared in only one dipteran GOBP. Interestingly, the motif patterns of PBPs were also different between the Lepidoptera and Hymenoptera. Similarly, motif patterns of lepidopteran and dipteran PBPs were analyzed by combining the PBP sequences of both lepidopteran and dipteran into a set of sequences. The order of the eight motifs in the Lepidoptera was 7-3-2-4-5-8-6-1 whereas it was 3-7-4-2-5-1-8-6 in the Hymenoptera (Figure 5). Furthermore, one PBP lacked one motif and two PBPs lacked five motifs in the Hymenoptera. These differences may imply functional differences of OBPs in different Orders. It should be noticed that the motifs found by MEME server are not comparable when different sets of sequences were used for analysis. Thus, it is not suitable to compare the motifs in different figures (figure 2, 3, 4, 5) since we used different input sequences.

### Phylogenetic analysis of OBPs and CSPs

**Figure 6:**
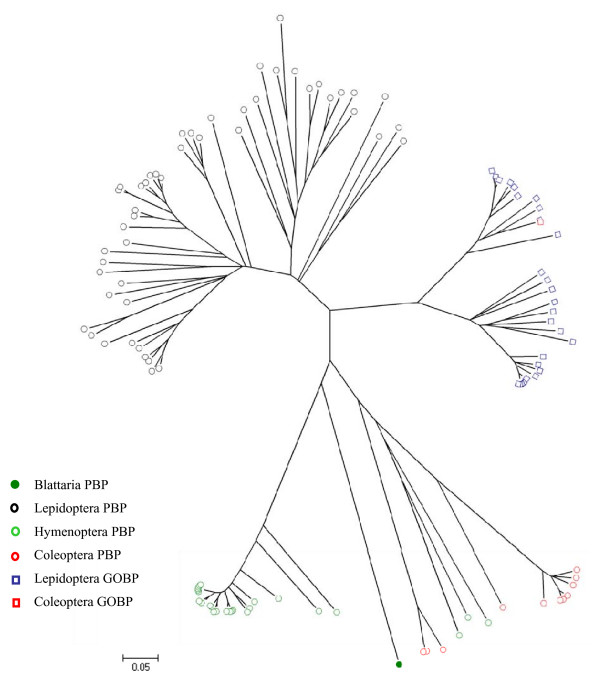
**The evolutionary tree of PBP and GOBP**. The tree was constructed by MEGA4.0 program with neighbor-joining phylogeny and the p-distances model. It was generated with 1000 bootstrap replications. Only the proteins originally named as PBP or GOBP were used in this analysis. The accession numbers of GOBPs and PBPs used in this analysis were listed in Additional File 6. The bootstrap values were given in Additional File 8-A.

**Figure 7:**
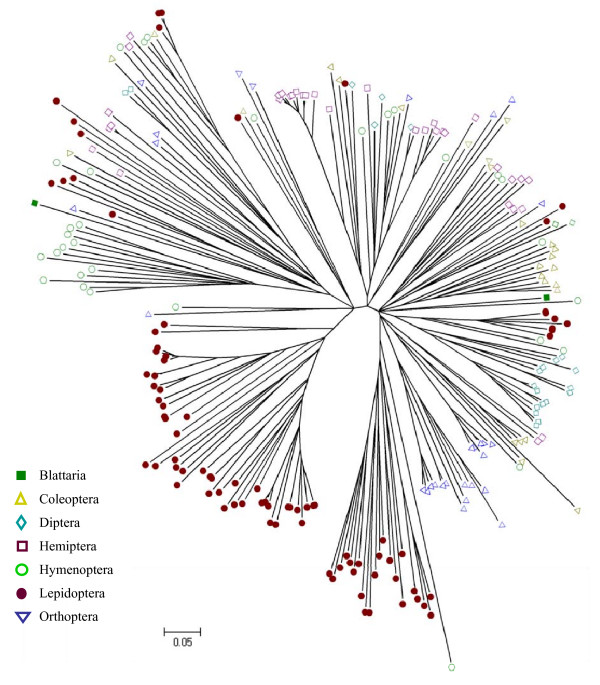
**The evolutionary tree of CSP**. The tree was constructed by MEGA4.0 program with neighbor-joining phylogeny and the p-distances model. It was generated with 1000 bootstrap replications. The accession numbers of CSPs used in this analysis were listed in Additional File 6. The bootstrap values were given in Additional File 8-B.

The neighbor-joining trees were inferred by MEGA4.0 using the p-distance amino acid model after 1000 bootstrap replicates [38]. In the evolutionary tree for GOBPs and PBPs, these two subfamilies were mainly clustered by Orders, indicating that most genes appeared after diversification of different Orders (Figure 6). This is consistent with the existence of an Order-specific motif-pattern as described above, suggesting that most GOBP and PBP genes have evolved recently. However, the situation is different for CSPs. Although lepidopteran CSPs were mainly clustered as an independent group, some of their CSPs are in the same clade with other Orders, suggesting that some CSPs are ancient, whereas others appeared after the diversification of Orders (Figure 7).

### Experimental validation of identified OBPs and CSPs

**Figure 8:**
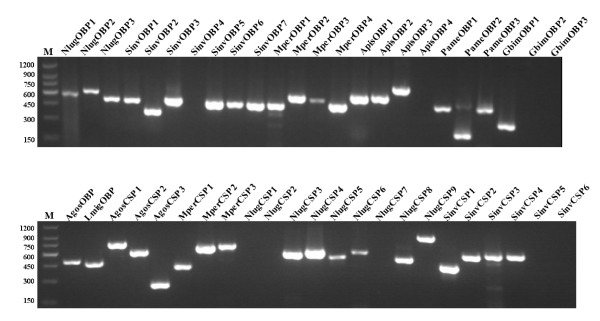
**RT-PCR validation of predicted OBPs and CSPs**. M: 150 bp DNA ladder. Primers used for the RT-PCR validation were listed in Additional File 7.

Most predicted OBPs or CSPs of full length were assembled from several ESTs. To validate the reliability of the computational pipeline, we randomly chose 26 OBPs from eight species and 22 CSPs from four species for RT-PCR validation. To cover a sequence that was as long as possible, the primers were designed at both ends of the transcripts assembled by the CAP3 software. As a result, 22 OBPs and 16 CSPs were successfully amplified by RT-PCR. The PCR results were confirmed by sequencing (Figure 8). Most validated OBPs and CSPs contains intact ORFs.

## Discussion

With only a few insect genomes sequenced, expressed sequence tags (EST) are a good resource for new gene discovery and expression profile analysis. As the cost of sequencing rapidly deceases, an abundance of insect ESTs has become available particularly in recent years, providing an opportunity to discover new OBPs and related genes at large-scale level. In this study, more than 100 new OBPs and CSPs were found from insect ESTs, suggesting that this approach is effective.

Although more than 10 OBP or CSP genes were found in some insects, less than five OBPs were identified in most species. Generally, there is no correlation between the number of identified genes and that of ESTs. Though some OBPs and CSPs are ubiquitous or expressed in non-sensory organs, both these two classes of proteins are believed to be abundant in the antennae and other chemosensory organs. Therefore, a high number of ESTs are not enough for finding many OBPs or CSPs if the ESTs were not from the chemosensory organs.

In agreement with previous reports[11], C-pattern and motif pattern analysis indicate that OBPs are more divergent than CSPs and suggests that OBP genes are still evolving. Zhou et al. used motif "C1 X6-8 C2 X16-21 C3 X2 C4 X3" to search CSP genes and successfully found 74 new genes[32]. In our work, the CSP motifs analyzed by Order are consistent with Zhou's results. Zhou et al. also analyzed the insect OBPs and obtained two motifs, "C1 X15-39 C2 X3 C3 X21-38 C4 X7-15 C5 X8 C6"for general OBP and "C1 X8-41 C2 X3 C3 X39-47 C4 X17-29 C4a X9 C5 X8 C6 X9-11 C6a" for Plus-C OBP[35]. Generally, the motifs analyzed by Order including new OBP genes found in our work are similar with Zhou's report. This proves the C-patterns of both OBP and CSP genes are highly conserved.

As most known PBP and GOBP genes have been identified in the Lepidoptera, we conducted a MEME motif analysis to compare the difference between these two subfamilies of OBP genes. Interestingly, all PBPs have an identical MEME motif pattern as 6-1-2-8-3-4-5-7, though they are more divergent than GOBPs at the protein-sequence level. GOBPs show four different motif patterns, with the most common one being 7-6-1-2-8-3-4-5. To the best of our knowledge, this is the first report of motif difference between GOBP and PBP subfamilies. This difference in the motif pattern might imply a functional difference between PBPs and GOBPs. Meanwhile, it also provides a hint that GOBP genes might have broad functions. Generally, PBPs bind and transport sex pheromones, while GOBPs are involved in sensing plant volatiles. Recent report by Zhou et al. proves that BmorGOBP2 in *B. mori *can also bind sex pheromone component (bombykol) [35]. Although sex pheromones in moths are species-specific, their chemical structures are similar, consisting of a hydrocarbon chain that contains an oxygenated functional group (ester, alcohol, aldehyde or epoxides) [39]. Thus, it is reasonable that PBPs from different insects have an identical motif. By contrast, GOBPs can bind both plant volatiles and sex pheromone, which display a broad diversity in chemical structures. We argued that this is the reason why GOBPs have divergent motif patterns.

In addition, we found that the C-patterns are similar, whereas the motif patterns are different among diverse Orders. We reasoned that C-pattern is the key structure of OBPs and CSPs, which should be highly conserved. But motif pattern fine-tune the functions of OBPs and CSPs, leading to the minor difference in binding sex pheromone or plant volatiles in different insect Orders.

## Conclusion

In conclusion, our results indicate that the computational pipeline we used in this study is efficient and reliable in identifying new OBP and CSP genes with insect EST resources. The large number of the newly found OBPs and CSPs in our study provides the basis for functional studies of these proteins. In addition, analysis of protein sequences showed that there is generally no major difference in C-patterns of OBPs or CSPs between different insect Orders, whereas conserved motif patterns are quite different between insect Orders and between the GOBPs and PBPs in Lepidoptera. Together with the evolutionary analysis, the results provide some new insights into the differentiation and evolution of insect OBPs and CSPs.

## Methods

### Insects

The cotton aphid (*Aphis gossypii*), peach aphid (*Myzus persicae*), brown plant hopper (*Nilaparvata lugens*) and pea aphid (*Acyrthosiphon pisum*) were collected from the campus of Nanjing Agricultural University. The Asiatic migratory locust (*Locusta migratoria*) was bought from an insect rearing factory in Shandong province, China. The American cockroach (*Periplaneta americana*) was provided by Professor Zhi-Kuan Jiang (Nanjing Institute of Military Medical Sciences). The red fire ant (*Solenopsis invicta*) was collected in Guangdong province with assistance of Professor Wen-Qing Zhang (Sun Yat-Sen University). The two-spotted cricket (*Gryllus bimaculatus*) was collected in Tianjin City, China.

### Data collection

Insect ESTs were downloaded from the dbEST [36] of NCBI http://www.ncbi.nlm.nih.gov/dbEST/ in March 2008. Sequences of known insect OBPs were obtained by searching the GenBank with the keywords "odorant-binding protein AND insecta NOT chemosensory protein NOT (Haemolymph juvenile hormone binding protein OR JHBP)". In total, 837 OBP sequences were downloaded, which covered all reported insect OBPs except for those from *Drosophila *species. At present, the genome sequences of 12 *Drosophila *species are available, of which hundreds of OBPs were identified. Because OBP genes share high similarities between different *Drosophila *species, only OBPs from *Drosophila melanogaster *were considered for analysis. Finally, 795 OBP sequences remained for further analysis after removing identical sequences. The non-redundant protein sequences (nr) were downloaded from the FTP server of NCBI. In total, 290 CSP sequences were retrieved from the UniProtKB [40,41].

### Computational pipeline for gene discovery

The computational pipeline is shown in Figure 1. The sequences of known OBPs and CSPs were used to search a local database of insect ESTs using the program TBLASTN [42] (version 2.2.17) by an e-value of 10.0. To get more information, the Blast hits were used as the queries to search the local EST database using the BLASTN [42] program (e-value = 1.0e-20). The ESTs meeting the criteria were collected. After removal of the identical sequences by perl scripts, the remaining sequences were assembled with CAP3 software (version date: 08/29/02) [43]. Then, the assembled sequences were used as queries to search against non-redundant protein sequences (nr) with the BLASTX program (default parameter) [42]. We kept those sequences whose blast hits of BLASTX are PBP_GOBP [44] or OS-D [10,12,45] family as putative OBP or CSP genes. The deduced protein sequences were further confirmed by searching the Pfam database with the default parameter (e-value = 1.0) [46].

Accession numbers of all OBP or CSP sequences used for C-Patten analysis, motif analysis and phylogenetic analysis are listed in the Additional File 6.

### C-Patten analysis

The protein sequences of OBPs and CSPs were aligned using ClustalX [47] (version 1.83) with default gap-penalty parameters to locate six or four conserved cysteines, and only those sequences with six (for OBP) or four (for CSP) conserved cysteines were used for C-pattern analysis. The number of amino acids between cysteines was counted separately.

### Motif analysis

According to the average length of known genes, the predicted ORFs with more than 120 amino acids (aa) for OBPs and 100 aa for CSPs were regarded as intact ORFs. All OBP and CSP sequences with intact ORF were used for motif discovery and pattern analysis. Parameters used for motif discovery were: minimum width = 6, maximum = 10, maximum number of motif to find = 8. Motif analysis was conducted by using MEME [48] (version 3.5.7) online server http://meme.sdsc.edu. The motifs identified in more than half of the input sequences with a p-value < 0.0001 were counted and viewed by WebLogo [49].

### Phylogenetic analysis

To improve the reliability, only those sequences covered the region of six cysteines (for OBP) or four cysteines (for CSP) were used in phylogenetic analysis. In total, 114 OBP and 224 CSP sequences were used. The protein sequences were aligned by ClustalX (version 1.83) with default gap-penalty parameters. The evolutionary trees were constructed based on consensus sequence by the MEGA4.0 [38] program with neighbor-joining [50] phylogeny using the p-distances model. An un-rooted tree was generated with 1000 bootstrap replications.

### RNA extraction and cDNA synthesis

The whole bodies of cotton aphids, peach aphids and pea aphids were used for RNA extraction, whereas only the heads with antennae of brown plant hoppers and red fire ants were collected. For the American cockroach, Asiatic migratory locust and two-spotted cricket, the antennae were dissected and used for RNA extraction. The collected tissues were fast-frozen in liquid nitrogen and kept at -70°C until further use. Total RNA was extracted by homogenizing antennae or other tissues in Trizol™ reagent (Invitrogen, Carlsbad, CA, USA) or E.Z.N.A.^® ^Total RNA Kit II (Omega) following the manufacturer's instructions. The cDNA template was synthesized with Oligo(dT)18 primer as anchor primers, using M-MLV reverse transcriptase (Invitrogen, Carlsbad, CA, USA) at 37°C for 50 min. The reactions were stopped by heating at 70°C for 15 min. Alternatively, we used AMV reverse transcriptase (Takara) at 42°C for 60 min, and stopped the reactions by cooling on ice for 5 min.

### RT-PCR

Gene specific primers across ORF of predicted OBP and CSP genes were designed using "Primer Premier 5.0" for RT-PCR validation. The sequences of these primers are listed in Additional File 7. PCR experiments were carried out in a PTC-200 (Bio-Rad, Waltham, MA, USA), and Touchdown PCR reactions were performed under the following conditions: 94°C for 3 min; 20 cycles at 94°C for 50 sec, 65°C for 1 min, and 72°C for 50 sec, with a decrease of the annealing temperature of 0.5°C per cycle. This was followed by 15 cycles at 94°C for 50 sec, 55°C for 1 min, and 72°C for 50 sec, and final incubation for 10 min at 72°C. The reactions were performed in 25 μl with 200-600 ng of single-stranded cDNA, 2.0 mM MgCl_2_, 0.5 mM dNTP, 0.4 μM for each primer and 1.25 U Taq polymerase or EX-Taq polymerase (Takara). PCR products were analyzed by electrophoresis on 1.5% w/v agarose gel in TAE buffer (40 mmol/L Tris-acetate, 2 mmol/L Na_2_EDTA·H_2_O) and the resulting bands were visualized with ethidium bromide. DNA purification was performed using the AxyPrep™ PCR Cleanup Kit (Axygen). Purified products were sub-cloned into a T/A plasmid using the pGEM-T easy vector system (Promega) following manufacturer's instructions. The plasmid DNA was used to transform into competent DH5a or Top10 cells. Positive clones were checked by restriction enzyme cleavage sites and PCR. Plasmid extraction was performed by E.Z.N.A.™ Plasmid Mini kit (Omega). The PCR products were sequenced by Bioasia (Shanghai, China).

## Authors' contributions

XY and ZL carried out the informatics works, and participated in manuscript writing; HP carried out the molecular biology experiments and participated in manuscript writing; FS helped in carrying out the informatics work; DS, ZY and LF conceived and designed the study; DS and LF are responsible for manuscript writing. All authors read and approved the final manuscript.

## Supplementary Material

Additional File 1 An Excel file with the name of "AF1-detail information of predicted OBP and CSP". Items in blue were previously reported and those in black were newly identified by present study. The OBP or CSP names that suffixed with same first number (such as GbimCSP8, GbimCSP8-1 and GbimCSP8-2) in the first column of the table were from a same sequence, therefore, the total items of OBP and CSP in this file are more than the numbers listed in Table 1. Click here for file

Additional File 2 A Text file named by "AF2- nucleotide sequence of identified OBP". Click here for file

Additional File 3 A Text file named by "AF3-protein sequence of identified OBP". Click here for file

Additional File 4 A Text file named by "AF4- nucleotide sequence of identified CSP". Click here for file

Additional File 5 A Text file named by "AF5- protein sequence of identified CSP". Click here for file

Additional file 6 An Excel file named by "AF6-accession numbers of OBP and CSP used for analysis". It shows the names and accession numbers of the proteins used in Figure 1, 2, 3, 4, 5, 6 &7 and Table 2 &3. The accession numbers for proteins identified in the present study are not available. Click here for file

Additional File 7 A Doc file named by "AF7-primers of OBPs and CSPs for RT-PCR validation". Click here for file

Additional File 8 A Doc file named by "AF8-evolutionary trees showing the bootstrap values". Click here for file
