# Supplementary material for: Large-scale identification of odorant-binding proteins and chemosensory proteins from expressed sequence tags in insects
Source: BMC Genomics. 2009 Dec 25;10:632. doi: 10.1186/1471-2164-10-632 (PMC2808328; doi:10.1186/1471-2164-10-632)
Supplement: Additional File 7 — A Doc file named by "AF7-primers of OBPs and CSPs for RT-PCR validation". [file 1471-2164-10-632-S7.doc]

**AF7 Primers of OBPs and CSPs for RT-PCR validation**

| Candidate OBPs and CSPs | Length of prediction | Length of PCR product | Result of PCR | Forward primer ( 5’ → 3’) | Reverse primer ( 5’ → 3’) | Genbank number |
| --- | --- | --- | --- | --- | --- | --- |
| AgosOBP | 496 | 496 | Amplified | gagcgaacaactgtcgtaaaatctt | aatgttcaaactcatactttccgtt | FJ215304 |
| NlugOBP1 | 620 | 620 | Amplified | ggttactggtctcagtctcgctatc | tgcctgtcgatttcctttatagaag | FJ215305 |
| NlugOBP2 | 675 | 678 | Amplified | ggcacagtg ctctcaataggc | tggtttcaaaggttttattcaaaca | FJ215306 |
| NlugOBP3 | 565 | 563 | Amplified | attaccctggttttcctatcgttg | ttttcatcacagcggttttcata | FJ215307 |
| ApisOBP1 | 809 | 695 | Amplified | aaattctccagtccgcaaaa | ccggaaaatatcattaaaatcctaa | FJ215320 |
| ApisOBP2 | 632 | 638 | Amplified | aaaaggaacacagctgaagtatggt | cgtgcatttgaatttttaatacaca | XM_001952488 |
| ApisOBP3 | 768 | 767 | Amplified | ttcgtcgacattatgaaggtatctg | cattttccgtccacatatcaagact | XM_001942687 |
| ApisOBP4 | 650 |  | Unamplified | actataccactgcggtctatgacaa | agttataggcgtacagcgttcaa |  |
| PameOBP1 | 498 | 497 | Amplified | ttcgtcgtctgtctgtcggtg | gatggtcacgtcgcaatcttg | FJ215311 |
| PameOBP2 | 258 | 250 | Amplified | gcagaaccaagatattttggaaagt | accgatgcatgttgaatgctata | FJ215312 |
| PameOBP3 | 493 | 489 | Amplified | cggcagtattcaaagaggaaaatc | tgtaagtacaggccatggttatcaa | FJ215313 |
| LmigOBP | 469 | 469 | Amplified | aattcgaattccgacatgaggac | atctcccttattctgacggttgc | FJ215322 |
| GbimOBP1 | 329 | 329 | Amplified | gaaggcaagtgctttcttggctg | gtagcaattcggcagaagtgggt | FJ215321 |
| GbimOBP2 | 495 |  | Unamplified | ctcactatagggaattcgacgtctag | tttattccccaatgcacacagtt |  |
| GbimOBP3 | 459 |  | Unamplified | gtcttcaagaagccacaaacagc | ctagtaagtgagatccccaccaaac |  |
| SinvOBP1 | 548 | 548 | Amplified | tgcaagcgaacgtacgacg | tttttctccgcgagcgaat | FJ215314 |
| SinvOBP2 | 413 | 416 | Amplified | tgtacggtccgacagaaatagtgt | ccgtatatttaactgtgtttattccgt | FJ215315 |
| SinvOBP3 | 562 | 562 | Amplified | tgaaactttcgtactttgtgctt | catcatcatgttctgcttctaca | FJ215316 |
| SinvOBP4 | 617 |  | Unamplified | acaatccctgatacaaaaggtacaac | aagtttggtttttggagttcattct |  |
| SinvOBP5 | 520 | 517 | Amplified | gtctaatgaacaggtttatctcggaa | accagggaaataaaatggagtctaa | FJ215317 |
| SinvOBP6 | 509 | 509 | Amplified | tgaacatctagtgctgtgcgc | tcttttcatttggttcttctg | FJ215318 |
| SinvOBP7 | 500 | 500 | Amplified | ggaagctcgagacaccagga | atcgtaacgtgagacgaggc | FJ215319 |
| MperOBP1 | 511 | 511 | Amplified | ttcaactgacagaacaccgaa | tggatggaatcaaaactgtatcgtt | FJ215308 |
| MperOBP2 | 621 | 622 | Amplified | actcagttttattctcaatccacgag | ttcctttgtacccaggatctatcag | FJ215309 |
| MperOBP3 | 570 | 570 | Amplified | tatacacttcgatcttaaacgcaaaa | ttaatagtaatttaaaggcgtacagcg | FJ215310 |
| MperOBP4 | 514 | 514 | Amplified | attctacagttcacaatcaaagtgcg | ctggttcgcttgactctcgtttc |  |
| AgosCSP1 | 742 | 742 | Amplified | agcagaattgcagtagtctgtgt | tgactgcttttatttcaaattaaat | FJ387486 |
| AgosCSP2 | 590 | 590 | Amplified | acagtaactcctctccgaatctcc | atcgacataatcagtcgtcacacat | FJ387487 |
| AgosCSP3 | 245 | 245 | Amplified | gatatatgtcaatgatggagaaaatc | aagtttttcgtaatcattgggac | FJ387488 |
| MperCSP1 | 392 | 390 | Amplified | cgcttttgagtacaatacattaaatca | tttgtagagtccttgggggtcat | FJ387489 |
| MperCSP2 | 640 | 640 | Amplified | ggcttaccaaatatcgtgtcttctt | catgcaactttgacgtttgagag | FJ387490 |
| MperCSP3 | 650 | 650 | Amplified | acacaggtcacccacaacaacac | tagacgattttcacattgcgaca | FJ387491 |
| NlugCSP1 | 655 |  | Unamplified | tttcacatctgataacgccattg | aacttcagactttcatccttgcg |  |
| NlugCSP2 | 592 |  | Unamplified | ctatctcaagtgtctgatggggg | tcaagattactgatgagacaccctg |  |
| NlugCSP3 | 540 | 540 | Amplified | attctttgcccaccagtttccat | tgaataggaaaggatttttagtggttg | FJ387492 |
| NlugCSP4 | 577 | 565 | Amplified | cgttcgatgtttctgatcgcagt | ctgatgaatgggaatatgggaag | FJ387493 |
| NlugCSP5 | 488 | 488 | Amplified | tttcgattgcagcaagatgaggt | acgcaggttgaaacacagagagc | FJ387494 |
| NlugCSP6 | 756 | 754 | Amplified | cgaccgtacctcagataactcag | gatgaaacttatcattaacagcctaga | FJ387495 |
| NlugCSP7 | 1163 |  | Unamplified | gtcattctgctgctaactacaactca | ttcacactcgaaaaatacattcact | FJ387496 |
| NlugCSP8 | 463 | 463 | Amplified | ttcttcactcatctaatcgttatcagt | tcgagtaaggttgaggagagttg | FJ387497 |
| NlugCSP9 | 794 | 792 | Amplified | ctaatcgtgagttgtgtgtgttgg | gtggttggttgatataggggaaa | FJ387498 |
| SinvCSP1 | 388 | 388 | Amplified | gctcgattaactgtatcgcgtta | ttcattattcatttttgttgccag | FJ387499 |
| SinvCSP2 | 507 | 507 | Amplified | tgtactatggctcgattaactgtattg | catagggtaccgtttacaaatactattt | FJ387500 |
| SinvCSP3 | 525 | 525 | Amplified | gctcgattgagtagtatcgcatt | gcttattacttccctttcccttc | FJ387501 |
| SinvCSP4 | 513 | 513 | Amplified | aactatggctcattaaatcgtatcg | tttccatctttcatactaccgtttac | FJ387502 |
| SinvCSP5 | 355 |  | Unamplified | gttgtctcactagtggttctggc | cattaaaatactttcaaggaattacga |  |
| SinvCSP6 | 479 |  | Unamplified | aatcgataccgtctcgctctttc | gtgaccggcgcgtttatattata |  |
