# Supplementary material for: Large-scale identification of odorant-binding proteins and chemosensory proteins from expressed sequence tags in insects
Source: BMC Genomics. 2009 Dec 25;10:632. doi: 10.1186/1471-2164-10-632 (PMC2808328; doi:10.1186/1471-2164-10-632)
Supplement: Additional File 8 — A Doc file named by "AF8-evolutionary trees showing the bootstrap values". [file 1471-2164-10-632-S8.doc]

Blattaria PBP

Lepidoptera PBP

Hymenoptera PBP

Coleoptera PBP

Lepidoptera GOBP

Coleoptera GOBP

**AF 8 Evolutionary trees showing the bootstrap values. A: PBP and GOBP evolutionary tree; B: CSP evolutionary tree**

**A**

**B**

Hemiptera

Hymenoptera

Lepidoptera

Orthoptera

Blattaria

Coleoptera

Diptera


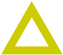

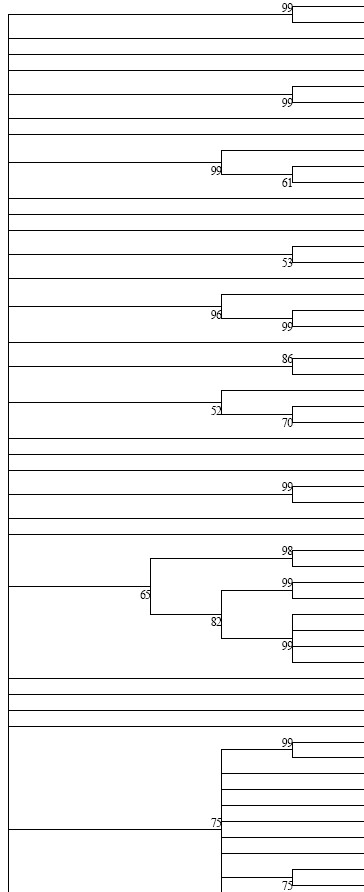

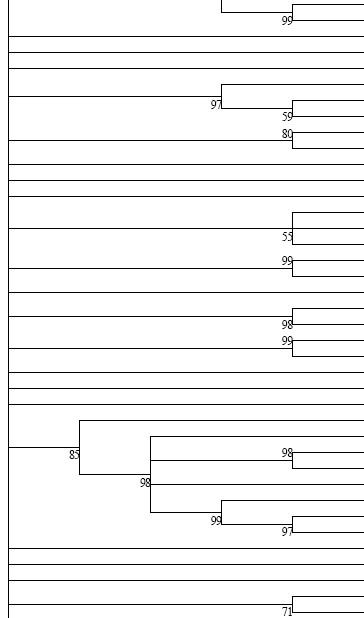

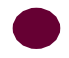

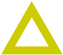

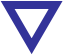

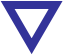

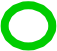

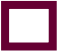

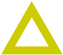

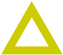

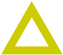

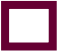

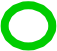

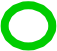

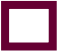

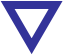

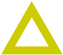

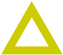

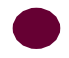

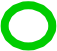

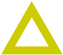

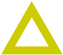

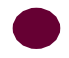

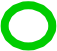

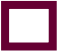

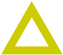

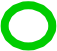

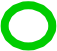

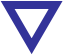

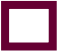

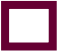

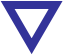

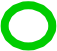

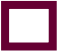

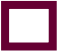

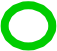

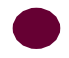

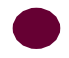

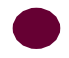

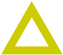

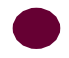

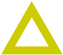

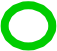

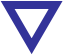

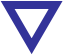

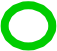

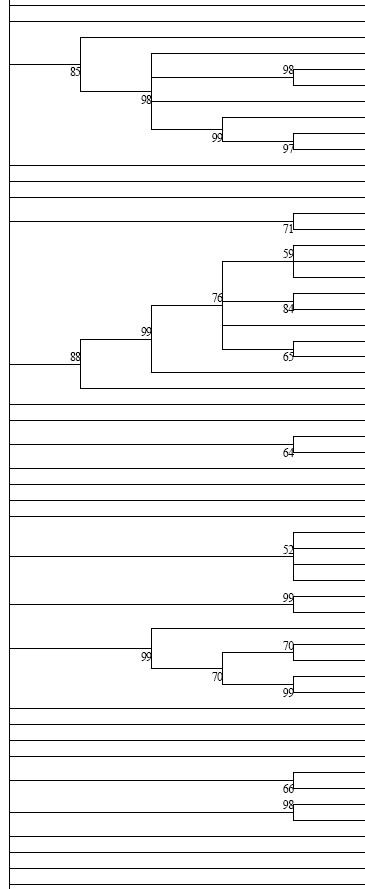

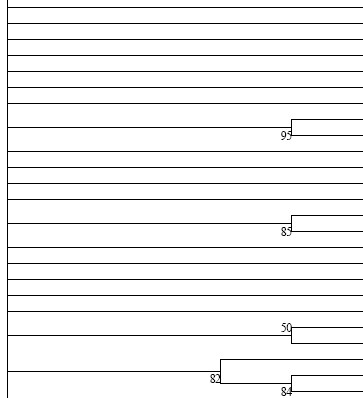

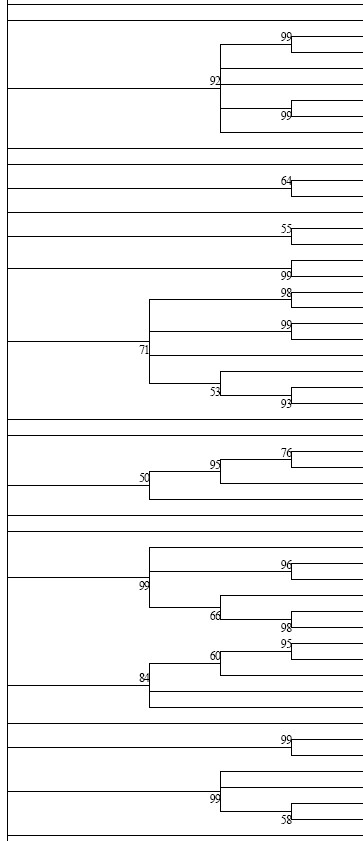

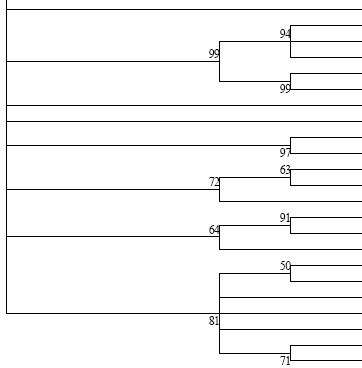

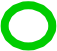

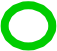

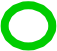

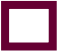

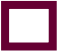

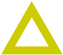

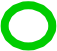

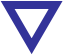

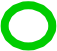

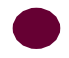

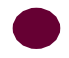


**3**

**2**

**1**
